# Spatial Variation and Source of Dissolved Heavy Metals in the Lancangjiang River, Southwest China

**DOI:** 10.3390/ijerph17030732

**Published:** 2020-01-23

**Authors:** Bin Liang, Guilin Han, Jie Zeng, Rui Qu, Man Liu, Jinke Liu

**Affiliations:** Institute of Earth Sciences, China University of Geosciences (Beijing), Beijing 100083, China; liangbin@cugb.edu.cn (B.L.); zengjie@cugb.edu.cn (J.Z.); qurui@cugb.edu.cn (R.Q.); lman@cugb.edu.cn (M.L.); liujinke@cugb.edu.cn (J.L.)

**Keywords:** natural processes, anthropogenic inputs, agricultural activities, water quality, Lancangjiang River

## Abstract

Dissolved heavy metals are not only the essential micronutrients, but also the toxic elements for human bodies. To investigate the heavy metal sources and assess the water quality of the Lancangjiang River, dissolved Cr, Ni, Cu, Zn, Mo, and Pb were detected in this study. The results show that dissolved Ni and Mo, Cr and Pb, and Cu and Zn were similarly distributed within the drainage basin. The correlation analysis exhibited that dissolved Ni and Mo had correlation with water parameter, and dissolved Cu was weakly correlated with Ni, indicating that they might be affected by natural processes. The principal component analysis explained 68.342% of the total variance for three principal components, of which dissolved Ni, Mo, and Cu were controlled by natural inputs; dissolved Cu and Cr were affected by anthropogenic activities; and dissolved Zn was influenced by agricultural activities in the downstream. The water quality showed that the water in upstream was worse than in midstream and downstream, and the whole drainage basin had water of excellent quality. Water within the drainage basin poses no risks to human bodies via daily diets and dermal routes. Dissolved Zn, Cu, and Mo occupied the major proportion of heavy metals transporting into the Mekong River. The agricultural inputs of dissolved Zn might pose potential risks to the Mekong River.

## 1. Introduction

Rivers play an important role in environmental cycles by dissolving and transporting heavy metals on Earth’s surface [1,2,3,4]. Annually, global rivers import 1 × 10^9^ tons of dissolved heavy metals into the ocean [1,5]. As is known, many heavy metals, such as Cr, Ni, Cu, Zn, Mo, Pb, etc., are also the essential elements in human bodies and cause severe diseases [6,7,8,9,10]. However, dissolved heavy metals would threaten human health if the concentrations were exorbitant [11,12,13,14,15]. For people living by rivers, the river water is the direct source of domestic water [16,17,18,19,20,21]. Therefore, it is significant to evaluate the environmental risks of dissolved heavy metals in river water.

The Lancangjiang River is the upper reach of the Mekong River (MR, the world’s seventh longest river) and originates from the Qinghai-Tibet Plateau (QTP) in southwest China [22,23]. Natural processes and anthropogenic activities largely release heavy metals into rivers [11,24]. Since the uplift of QTP, global weathering has accelerated under the Asian monsoon climate [25,26,27,28,29,30,31,32], and may result in the increase of dissolved heavy metals in river water. In addition, with the development of the Lancangjiang River basin in recent years, human activities have increased greatly [33,34,35]. Based on the current situations of the Lancangjiang River, the amount of heavy metals in river water may have much more possibilities to threaten the environment. However, it is hard to constrain the sources of dissolved heavy metals in river water due to the complicated geological environment and human activities. Multivariate statistical methods, including correlation analysis and principal component analysis (PCA), are some of the most effective ways to identify the sources of heavy metals in river water [36,37,38,39], because they can better explain the water quality and possible sources affecting the study area by identifying hidden relations between variables and reducing complex chemical datasets to a small number of factors without much information loss [8,11].

In this study, six dissolved heavy metals (Cr, Ni, Cu, Zn, Mo, and Pb) in the Lancangjiang River water were measured to investigate the spatial distribution of dissolved heavy metals, identify their sources, and assess the water quality of the Lancangjiang River water. Moreover, this study also evaluated the dissolved heavy metal fluxes, aiming to assess the environmental impact on the Mekong River. The study was applied to enhance water management efficiency and prevent hazardous metal contamination to human health.

## 2. Materials and Methods

### 2.1. Study Area

**Figure 1:**
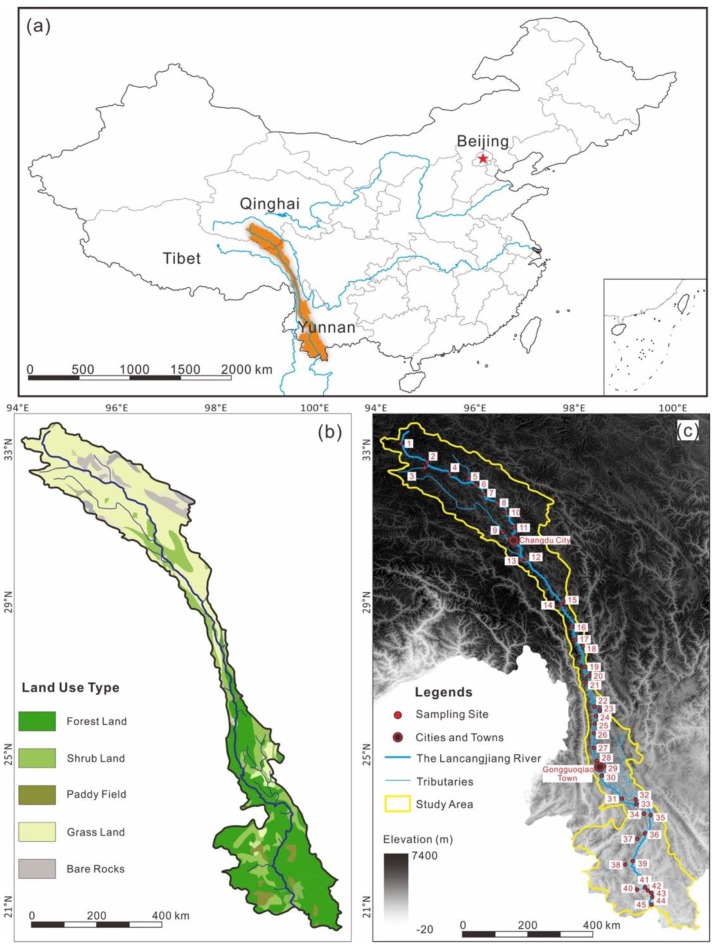
Map of study area: (**a**) location of the Lancang River; (**b**) sampling sites in the Lancang River, where Digital Elevation Model (DEM) data are from Resource and Environment Data Cloud Platform (http:/resdc.cn/data.aspx?DATAID=284); and (**c**) land use map of the Lancangjiang River basin, where land use data are from OSGeo (http:/osgeo.cn/map/m0409).

The Lancangjiang River locates within the latitude of 21°75′–33°81′ N and the longitude of 94°40′–101°15′ E, with a total length of 2160 km, an elevation difference of 4583 m, and an area about 168,000 km^2^ of the whole drainage basin [22,25]. The Lancangjiang River flows through Qinghai Province, Tibet Autonomous Region, and Yunnan Province in southwest China (Figure 1a). The Lancangjiang River is divided into three parts: (i) the upstream: from the source to Changdu City (Figure 1c), with an average elevation of more than 4000 m [40]; (ii) the midstream: from Changdu City to Gongguoqiao Town in Yunnan Province, characterized by large altitude differences, rapid water flows, and small population; and (iii) the downstream: from Gongguoqiao Town to Vietnam (Figure 1c), with less change of terrains and increase of tributaries and population [25].

The Lancangjiang River covers six climatic zones from north to south: frigid zone, cold temperate zone, temperate zone, warm temperate zone, subtropical zone, and tropical zone [33,34]. The average annual precipitation in the Lancangjiang River basin is more than 1200 mm, with <800 mm in the upstream, 800–1100 mm in the midstream, and >1100 mm in the downstream [34,41]. From the land-use map (Figure 1b), grassland and bared rocks are distributed in the north, while forest land and a little proportion of paddy land are distributed in the south, and shrub land spreads within the whole drainage basin. The land used by human activities are focused in the downstream [40].

### 2.2. Sampling

Samples were collected from downstream to upstream from 18 July to 7 August 2019. All sampling instruments were cleaned indoor and rinsed three times with river water in the field. Water samples were collected using a sampler, then filtered through a 0.22-µm filter membrane, stored in pre-cleaned high-density polyethylene (HDPE) plastic bottles, and labeled LCJ-1–45, where LCJ-1–11 belong to the upstream, LCJ-12–28 locate within the midstream, and LCJ-29–45 are from the downstream (Figure 1c). The Sampling coordinates are given in Table 1. For subsequent elemental measurement, the filtrate was acidified to pH < 2 using ultra-pure concentrated nitric acid. The collected samples were stored at 4 °C before analysis. Water temperature, pH, total dissolved solid (TDS), and dissolved oxygen (DO) were measured in situ using a YSI water quality monitoring meter (Xylem Inc., Yellow Spring, OH, USA).

### 2.3. Measurement of Dissolved Heavy Metals

The concentrations of heavy metals (Cr, Ni, Cu, Zn, Mo, and Pb) were determined by a high resolution inductively coupled plasma mass spectrometer (ICP-MS, Element 2XR, Thermo Fisher Scientific, Waltham, MA, USA) at State Key Laboratory of Geological Processes and Mineral Resources, China University of Geosciences (Beijing), coupled with an Elemental Scientific PFA nebulizer with a flow rate of 100 µL/min. Before analysis, 0.8 μg/L indium (In) was added into water solutions as the internal standard to monitor the signal shift. The Eppendorf pipettes (Eppendorf Corp., Hamburg, Germany) were calibrated in advance for accurately transferring water samples and standard solution, and the volume error was ±2%. The instrumental precision was tested by measuring a digested solution of the external standard BCR-2 (United States Geological Survey, Denver, CO, USA) between every 10 samples, and the analytical error was within ±5%. Method precision was also controlled by determining replicate samples between every 10 samples, and the relative standard deviations (RSDs) were ±4.3%.

### 2.4. Statistical Methods and Water Quality Assessment

The statistical analysis for correlation analysis and PCA was processed by SPSS 25.0 (IBM Corporation, Armonk, NY, USA). The Pearson correlation matrix was exported to explain the relations and interactions between each pair of dissolved heavy metals in the Lancangjiang River. The principal components (PCs) were investigated to explore the possible sources of dissolved heavy metals by a dimensionality reduction technique. Before conducting PCA, the concentrations of dissolved heavy metals were standardized by z-scale transformation and the adequacy and suitability of dataset was examined by the Kaiser–Meyer–Olkin (KMO) and Bartlett’s sphericity test in order to avoid numerical ranges of the original variables.

### 2.5. Water Quality Index

The water quality index (WQI) is one of the most comprehensive tools to present the quality of river water. It is calculated as follows:

WQI = Σ [W_i_ × (C_i_/S_i_)] × 100, (1)

where W_i_ represents the weight of each dissolved heavy metals and is obtained from the eigenvalues for each PC result; C_i_ represents the concentrations of dissolved heavy metals at each sampling site; and S_i_ is the drinking water guidelines recommended by the China GB 5749-2006 [42]. The water quality is categorized into five classes: (i) excellent water quality (0 ≤ WQI < 50); (ii) good water quality (50 ≤ WQI < 100); (iii) poor water quality (100 ≤ WQI < 200); (iv) very poor water quality (200 ≤ WQI < 300); and (v) water unsuitable for drinking (WQI ≥ 300) [9,11].

### 2.6. Hazard Index and Hazard Quotient

The hazard index (HI) and hazard quotient (HQ) are parameters to assess the toxicity of dissolved heavy metals in water. The HQ is the ratio between exposure via individual pathways and reference dose and the HI is the sum of the His from both pathways. When HI is smaller than 1, there is no deleterious effects on human health; however, when HI is larger than 1, non-carcinogenic risks or adverse effects on human health exist. The HQ and HI are calculated as follows:

ADD_ingestion_ = (C_w_ × IR × EF × ED)/(BW × AT), (2)

ADD_dermal_ = (C_w_ × SA × K_p_ × ET × EF × ED × 10^−3^)/(BW × AT), (3)

HQ = ADD/RfD,(4)

RfD_dermal_ = RfD × ABS_GI_, (5)

HI = ΣHQs,(6)

where ADD_ingestion_ and ADD_dermal_ represent the average daily dose via ingestion and dermal routes, respectively (μg/kg/day); C_w_ represents the mean concentration of each dissolved heavy metal in water (μg/L); BW is the average weight of human (70 kg for adults and 15 kg for children); IR is the ingestion volume per day (2 L/day for adults and 0.64 L/day for children); EF represents the exposure frequency (350 days/year); ED is the exposure period (30 years for adults and 6 years for children); AT is the average time (=ED × 365 days/year); SA defines the exposed skin area (18,000 cm^2^ for adults and 6600 cm^2^ for children); ET is the exposure time (0.58 h/day for adults and 1 h/day for children); K_p_ represents dermal permeability coefficient in water (cm/h); RfD is the reference dose (μg/kg/day); and ABS_GI_ is the gastrointestinal absorption factor. The reference values were obtained from the United States Environmental Protection Agency [43].

### 2.7. Dissolved Heavy Metal Fluxes

The Lancangjiang River flows southwards into the Mekong river. The fluxes of dissolved heavy metals into the Mekong River were estimated on the basis of the discharge data. The flux of each dissolved heavy metals was calculated using the following equation:

flux (tons/year) = Q_A_ × C_A_, (7)

where Q_A_ is average annual water discharge (2180 m^3^/s) [40] and C_A_ is the average concentration of dissolved heavy metals in downstream, where the terrain is relatively plain, the sampling sites are more dense, the variations of element concentration are small, and the Lancangjiang River connects the Mekong River.

## 3. Results

### 3.1. Physicochemical Parameters

**Figure 2:**
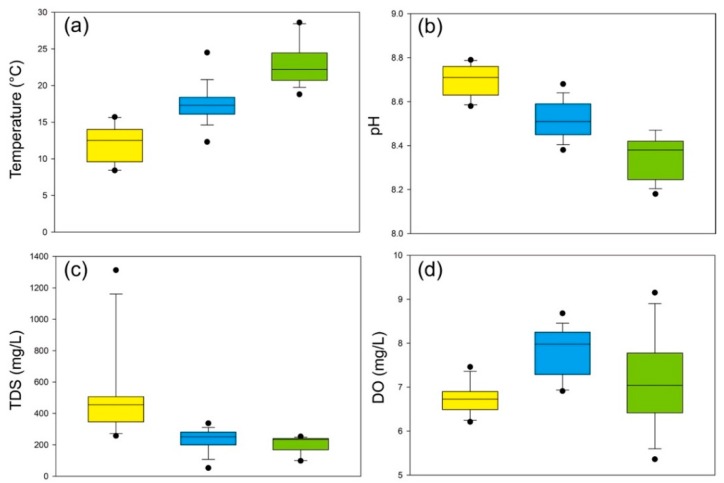
Boxplots of the chemical parameters of the Lancangjiang River: (**a**) temperature; (**b**) pH; (**c**) total dissolved solid (TDS); (**d**) dissolved oxygen (DO).

The water parameters are listed in Table 1 and their boxplots are displayed in Figure 2. The water temperature of the whole river has a large variation from 8.4 to 28.6 °C (Figure 2a). The river water exhibits alkaline with the pH values ranging from 8.2 to 8.8 with decreasing trend from upstream to downstream (Figure 2b), and alkaline river water may be conducive to the absorption and oxidization of dissolved heavy metals [44]. The TDS varies largely with the values of 52.7–1313.0 mg/L, with the peak values in the upstream (Figure 2c). Except for LCJ-3, the Lancangjiang River water is categorized as basically fresh water (TDS < 1000 mg/L). The DO ranges from 5.4 to 9.2 mg/L with relatively high values in the midstream (Figure 2d).

### 3.2. Concentrations of Dissolved Heavy Metals

The concentrations of dissolved heavy metals are reported in Table 2. The selected dissolved heavy metals are low concentrated in the Lancangjiang River. Dissolved Mo was the most abundant heavy metal in the river water, with the mean value >1 µg/L (0.25–3.06 µg/L). The other elements are less abundant with the mean values under 1 µg/L, and dissolved Cr, Ni, Cu, Zn, and Pb have the values of 0–0.69 µg/L, 0.05–3.28 µg/L, 0.05–2.80 µg/L, 0.09–4.81 µg/L, and 0.09–0.53 µg/L, respectively.

**Figure 3:**
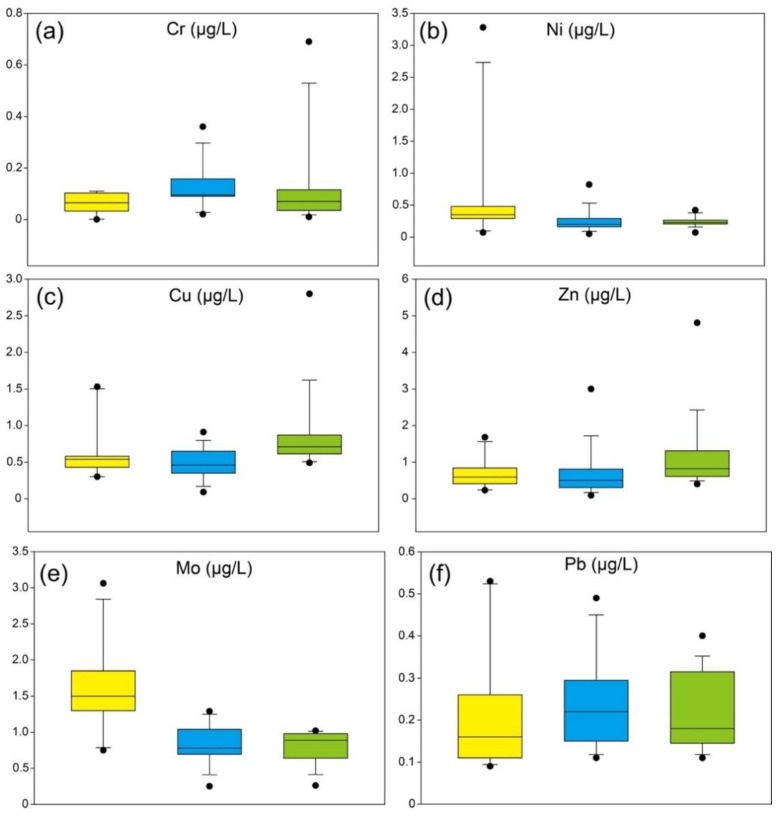
Boxplots of dissolved heavy metals in the Lancangjiang River: (**a**) Cr; (**b**) Ni; (**c**) Cu; (**d**) Zn; (**e**) Mo; (**f**) Pb.

The boxplots of dissolved heavy metals are displayed in Figure 3. Dissolved Ni and Mo are similar in spatial distribution, with the highest values in the upstream and lowest values in the midstream. The similarity was also shown in the distribution of dissolved Cr and Pb, with the highest mean values in the midstream and lowest values in the upstream. Dissolved Cu and Zn are abundant in the downstream and depleted in the upstream.

Compared with the mean concentrations of dissolved heavy metals in world’s major rivers (Table 2), dissolved Mo has a similar value compared with the upper Mississippi River (1.11 µg/L), dissolved Ni is similar to the values in two Chinese major rivers, with the values of 0.2 µg/L in the Yangtze River and 0.30–0.59 µg/L in the Yellow River, implying dissolved Ni might be affected by natural factors. Dissolved Cr and Cu are lower than those in the Yangtze River, Yellow River, and upper Mississippi River. Dissolved Zn is higher than the Zn concentration in the Amazon River. Dissolved Pb is higher than world’s rivers, and higher concentration of dissolved Pb may be affected by the Pb ore in the midstream, the largest Pb ore deposit in Asia [45].

## 4. Discussion

### 4.1. Correlation Analysis

To investigate the relationships between the physicochemical parameters and dissolved heavy metals and the interrelationships between each pair of dissolved heavy metals, the Pearson correlation matrix are displayed in Table 3. Among the metals, dissolved Mo and Ni were affected by water parameters at different degrees (Mo, *p* < 0.01; Ni, *p* < 0.05), indicating the influence of natural processes; dissolved Cu was weakly correlated with Ni (*p* < 0.05)); dissolved Zn was negatively correlated with pH (*p* < 0.05), indicating that Zn is more likely to be dissolved under weak acidic conditions; and the other elements had no significant correlations with each other and were weakly correlated with the water parameters, indicating that they had large chance to be from various anthropogenic activities, such as the discharge of industrial and domestic sewage, mining activities, construction of hydropower plants, etc. [8].

### 4.2. Concentrations of Dissolved Heavy Metals

The sources of dissolved heavy metals were identified using the PCA method, and the results are shown in Table 4. The results of KMO and Bartlett’s test were 0.390 and 39.962 (df = 15, *p* < 0.01), respectively, and the significance of the test is <0.001, indicating that PCA was effective in reducing dimensionality. Three principal components (PCs) were extracted using a varimax rotation method by being Kaiser-normalized, with the eigenvalues exceeding 1, and explaining 68.342% of the total variance. The PC loadings were classified into three groups: strong, moderate, and weak, with the loading values of >0.75, 0.75–0.50, and 0.50–0.30, respectively [46].

PC1, explaining 31.342% of the total variance, had strong loadings of Ni (0.880) and Mo (0.855) and a weak loading of Cu (0.478). Based on the correlation analysis, dissolved Mo and Ni were similarly correlated with temperature and pH, and dissolved Cu was also weakly correlated with Ni, indicating these elements were affected by natural processes. In addition, dissolved Mo was also strong correlated with TDS, and the concentrations of dissolved Mo and Ni increased with the pH and decreased with the temperature, suggesting dissolved heavy metals of PC1 might originated from the upstream of the Lancangjiang River, based on the distribution of water parameters (Figure 2). PC2 explained 20.277% of the total variance and had a moderate loading of Cr (0.634) and a weak loading of Cu (0.458). Dissolved Cr and Cu were reported to be affected by anthropogenic activities, such as industrial discharges and domestic wastes [47]. Additionally, they showed no significant correlation with water parameters, suggesting that PC2 was controlled by anthropogenic sources. PC3 was affected by the enrichment of dissolved Zn, with 16.723% of the total variance. The concentration of dissolved Zn had a negative correlation with pH values, and had no relationship with other parameters, implying that the anthropogenic inputs was more than natural inputs. Zn is one of the most common micronutrients in fertilizers and pesticides and has higher values (Figure 3d) in the downstream where agricultural activities increase (Figure 2b), and, thus, PC3 was attributed to agricultural inputs.

### 4.3. Water Quality Assessment

**Figure 4:**
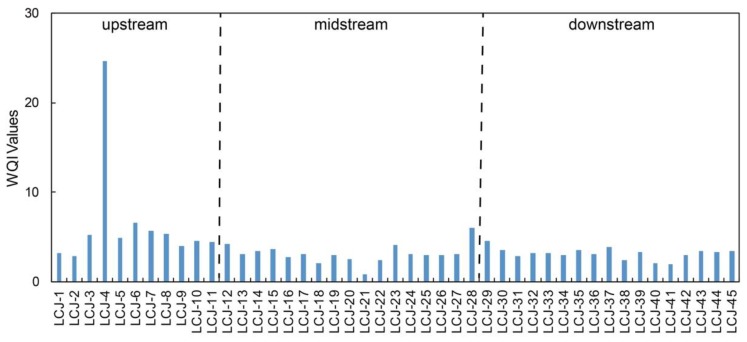
The water quality index for the water samples in the Lancangjiang River.

The water quality was assessed using WQI values calculating based on the weights of dissolved Cr, Ni, Cu, Zn, Mo, and Pb (Table 5), but the weight of dissolved Pb was unavailable because there are no loadings of Pb for any PCs. The results of WQI are shown in Figure 4. Water samples within the drainage basin had an excellent quality with WQI values under 50, indicating these dissolved heavy metals posed no health risks to human health. However, the water quality in the upstream was worse than in midstream and downstream, which may be attributed to the lack of water management due to the sparse population [33,34,40]. The high WQI value at site LCJ-4 might be affected by largely natural inputs due to the high concentration of dissolved Ni and Mo.

### 4.4. Health Risk Assessment

**Figure 5:**
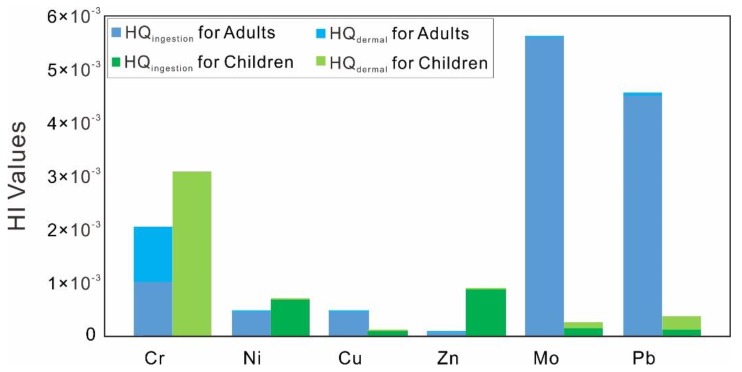
The hazard index values for the water samples in the Lancangjiang River.

The calculation for HQ and HI values is given in the Supplementary Materials (Table S1), and their values are also illustrated in Figure 5. In general, HQ_ingestion_ and HQ_dermal_ for both adults and children were smaller than 1, suggesting that no health effects and carcinogenic concern via daily intake and dermal absorption exist for human health. The HI values also exhibited that all dissolved heavy metals posed non-carcinogenic risks to human bodies. Meanwhile, dissolved Cu, Ni, and Pb had greater influence on adults than on children, and dissolved Cr preferred to be absorbed through dermal routes. Although all the dissolved heavy metals were within the values of no health risks, the discharge of dissolved Cr, Mo, and Pb had higher HI values and should be given more attention in the case of unnecessary health risks.

### 4.5. Elemental Fluxes to the Mekong River

**Figure 6:**
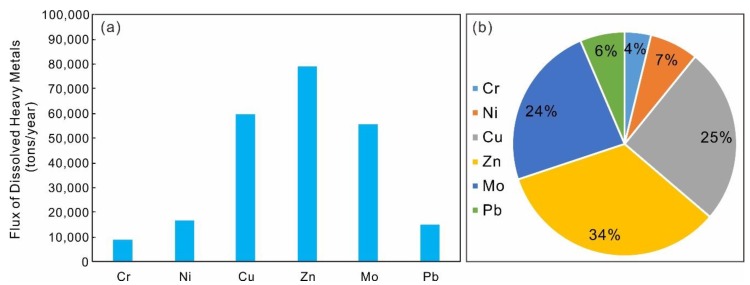
The flux of dissolved heavy metals in the Lancangjiang River: (**a**) the flux of dissolved heavy metals; (**b**) the proportion of each dissolved heavy metal flux.

Based on the average discharge data of the Lancangjiang River, the flux of each dissolved heavy metal is shown in Figure 6. Annually, the Lancangjiang River transported dissolved Cr, Ni, Cu, Zn, Mo, and Pb into the Mekong River, with the amounts of 8.93 × 10^3^, 1.65 × 10^4^, 5.98 × 10^4^, 7.91 × 10^4^, 5.57 × 10^4^, and 1.51 × 10^4^ tons/year, respectively (Figure 6a). Among the dissolved heavy metals, dissolved Zn, Cu, and Mo occupied the main proportion, with the percentage of 34%, 25%, and 24%, respectively (Figure 6b). Considering that Zn from agricultural activities were concentrated in the downstream, it might contribute to the Mekong River, pose potential risks, and needs more attention.

## 5. Conclusions

Based on data of water physicochemical parameters and dissolved heavy metals in the Lancangjiang River water, and multivariate statistical analysis, such as correlation analysis and PCA, the following conclusions were drawn regarding the sources of dissolved heavy metals and water quality of the Lancangjiang River:

(1) Dissolved Ni and Mo were similar in spatial distribution, the distribution of dissolved Cr and Pb were also similar, and dissolved Cu, and Zn are abundant in the downstream and depleted in the upstream.

(2) The correlation matrix showed that dissolved Ni and Mo had correlation with water parameter, and dissolved Cu were weakly correlated with Ni, indicating that they might be affected by natural processes.

(3) The PCA results explain that dissolved Ni, Mo, and Cu were controlled by natural inputs, dissolved Cu and Cr were affected by anthropogenic activities, and dissolved Zn was influenced by agricultural activities in the downstream.

(4) The water quality of the drainage basin is excellent, but the water in upstream was worse than in midstream and downstream. The water within the drainage basin poses no risks to human health based on the HI and HQ values.

(5) Dissolved Zn, Cu, and Mo were the main heavy metals transporting into the Mekong River. Dissolved Zn from agricultural activities contributed a large amount to the Mekong River and poses potential risks, needing more attention.

## Figures and Tables

**Table 1:** The sampling coordinates and water parameters obtained from field works.

	Longitude	Latitude	Temperature	pH	TDS	DO
	(°E)	(°N)	°C		mg/L	mg/L
LCJ-1	94.402991	33.456057	11.5	8.71	346.5	6.21
LCJ-2	94.595065	33.215437	8.6	8.74	257.4	6.90
LCJ-3	94.591304	33.211587	8.4	8.72	1313.0	6.87
LCJ-4	95.091903	32.975173	9.6	8.76	487.5	6.97
LCJ-5	95.545432	32.858943	13.4	8.67	455.0	6.40
LCJ-6	95.549912	32.844761	12.5	8.63	552.5	6.76
LCJ-7	96.150953	32.562826	12.2	8.79	507.0	6.72
LCJ-8	96.562116	32.140811	13.8	8.68	455.0	6.73
LCJ-9	97.120279	31.163799	14.0	8.78	330.9	6.49
LCJ-10	97.070229	31.712121	15.7	8.58	360.1	6.53
LCJ-11	97.217497	31.371264	15.4	8.61	353.6	7.46
LCJ-12	97.383187	30.721746	16.0	8.53	337.4	6.91
LCJ-13	97.353670	30.734584	15.2	8.55	194.4	6.94
LCJ-14	98.350127	29.639878	16.6	8.63	304.2	7.75
LCJ-15	98.367690	29.659875	16.2	8.38	204.8	7.11
LCJ-16	98.609130	29.088158	17.9	8.44	297.7	7.98
LCJ-17	98.788479	28.553069	17.9	8.56	293.8	7.32
LCJ-18	98.917709	28.473685	12.3	8.49	137.8	7.73
LCJ-19	98.921009	28.078747	16.9	8.49	267.8	8.68
LCJ-20	99.047241	27.709256	17.2	8.51	254.8	8.40
LCJ-21	99.000295	27.653755	15.9	8.62	52.7	8.31
LCJ-22	99.088791	27.355468	17.3	8.62	250.3	8.17
LCJ-23	99.130126	27.348985	19.9	8.68	120.9	7.26
LCJ-24	99.170807	27.104086	19.7	8.46	257.4	8.11
LCJ-25	99.188900	26.868327	24.5	8.44	251.6	7.83
LCJ-26	99.147854	26.476630	18.1	8.53	258.1	7.98
LCJ-27	99.126576	26.085784	18.4	8.47	241.8	8.38
LCJ-28	99.236585	25.752749	18.4	8.41	232.1	8.19
LCJ-29	99.373223	25.630842	22.7	8.40	202.2	7.18
LCJ-30	99.308713	25.423561	18.8	8.47	233.4	8.84
LCJ-31	99.866501	24.783772	23.2	8.35	234.0	9.15
LCJ-32	100.157401	24.826969	24.1	8.40	238.6	8.09
LCJ-33	100.099940	24.667684	20.0	8.24	247.7	7.68
LCJ-34	100.486163	24.528619	25.1	8.39	98.2	6.71
LCJ-35	100.497481	24.533862	20.7	8.38	252.9	7.39
LCJ-36	100.380259	23.991530	21.6	8.40	245.7	7.03
LCJ-37	100.103179	23.973999	22.0	8.46	129.4	6.93
LCJ-38	100.180689	23.544902	28.6	8.27	156.0	7.04
LCJ-39	100.171253	23.559853	22.4	8.18	239.9	7.15
LCJ-40	100.118367	22.626470	24.8	8.47	100.8	7.02
LCJ-41	100.396296	22.667391	28.4	8.44	181.4	7.87
LCJ-42	100.467455	22.591897	20.6	8.21	237.3	5.66
LCJ-43	100.580795	22.497485	20.7	8.24	241.2	5.36
LCJ-44	100.802062	22.015849	22.1	8.25	224.7	6.12
LCJ-45	100.922704	21.851740	22.2	8.38	236.0	5.92

**Table 2:** Concentrations and statistics of dissolved heavy metals in the Lancangjiang River and world’s major rivers.

	Cr	Ni	Cu	Zn	Mo	Pb
	μg/L	μg/L	μg/L	μg/L	μg/L	μg/L
Upstream						
LCJ-1	0.00	0.21	1.40	0.28	0.93	0.11
LCJ-2	nd^1^	0.07	0.44	0.47	1.38	0.16
LCJ-3	0.11	0.48	0.57	1.09	1.30	0.21
LCJ-4	0.06	3.28	1.53	0.84	3.06	0.21
LCJ-5	0.04	0.29	0.30	0.66	1.81	0.12
LCJ-6	0.01	0.54	0.58	0.66	1.97	0.16
LCJ-7	0.11	0.43	0.54	0.53	1.75	0.11
LCJ-8	0.04	0.35	0.56	0.59	1.85	0.26
LCJ-9	0.07	0.44	0.31	0.41	0.75	0.50
LCJ-10	0.10	0.30	0.43	1.68	1.50	0.53
LCJ-11	0.08	0.29	0.52	0.23	1.49	0.09
min	nd	0.07	0.30	0.23	0.75	0.09
max	0.11	3.28	1.53	1.68	3.06	0.53
mean	0.05	0.61	0.65	0.68	1.62	0.22
SD	0.04	0.90	0.42	0.41	0.61	0.15
Midstream	Cr	Ni	Cu	Zn	Mo	Pb
	μg/L	μg/L	μg/L	μg/L	μg/L	μg/L
LCJ-12	0.09	0.33	0.53	0.50	1.24	0.30
LCJ-13	0.17	0.36	0.29	0.19	0.45	0.12
LCJ-14	0.11	0.20	0.43	0.36	1.21	0.17
LCJ-15	0.36	0.15	0.77	0.24	1.29	0.11
LCJ-16	0.02	0.15	0.41	0.37	1.06	0.49
LCJ-17	0.09	0.20	0.42	0.71	1.02	0.16
LCJ-18	0.16	0.10	0.19	3.00	0.71	0.14
LCJ-19	0.09	0.19	0.46	1.40	0.94	0.21
LCJ-20	0.11	0.17	0.45	0.90	0.78	0.22
LCJ-21	0.03	0.05	0.26	0.83	0.25	0.35
LCJ-22	0.06	0.18	0.53	0.25	0.68	0.26
LCJ-23	0.27	0.46	0.91	0.79	0.51	0.13
LCJ-24	0.10	0.24	0.58	0.73	0.85	0.26
LCJ-25	0.09	0.25	0.68	0.39	0.77	0.44
LCJ-26	0.09	0.24	0.62	0.38	0.76	0.25
LCJ-27	0.15	0.21	0.68	0.51	0.89	0.20
LCJ-28	nd	0.82	0.09	0.09	0.75	0.29
min	nd	0.05	0.09	0.09	0.25	0.11
max	0.36	0.82	0.91	3.00	1.29	0.49
mean	0.12	0.25	0.49	0.69	0.83	0.24
SD	0.09	0.17	0.21	0.68	0.28	0.11
Downstream	Cr	Ni	Cu	Zn	Mo	Pb
	μg/L	μg/L	μg/L	μg/L	μg/L	μg/L
LCJ-29	0.07	0.42	2.80	0.88	0.74	0.21
LCJ-30	0.06	0.30	0.90	0.66	0.83	0.18
LCJ-31	0.03	0.18	0.84	0.71	0.89	0.14
LCJ-32	0.01	0.23	0.80	1.21	0.97	0.18
LCJ-33	0.02	0.25	0.79	1.41	0.86	0.15
LCJ-34	0.49	0.23	0.83	1.06	0.52	0.14
LCJ-35	0.07	0.28	0.71	0.72	0.95	0.11
LCJ-36	0.11	0.19	0.62	0.54	0.97	0.40
LCJ-37	0.69	0.37	0.61	0.82	0.54	0.25
LCJ-38	0.08	0.22	1.01	0.56	0.45	0.30
LCJ-39	0.08	0.23	0.70	4.81	0.97	0.33
LCJ-40	0.26	0.22	0.49	1.83	0.26	0.15
LCJ-41	0.03	0.07	0.69	0.51	0.80	0.32
LCJ-42	0.04	0.19	0.53	0.40	0.99	0.17
LCJ-43	0.04	0.22	1.33	1.53	1.02	0.12
LCJ-44	0.12	0.23	0.66	1.20	1.01	0.31
LCJ-45	0.07	0.25	0.51	0.76	1.00	0.34
min	0.01	0.07	0.49	0.40	0.26	0.11
max	0.69	0.42	2.80	4.81	1.02	0.40
mean	0.13	0.24	0.87	1.15	0.81	0.22
SD	0.18	0.08	0.54	1.02	0.23	0.09
Lancangjiang River River	0.11	0.33	0.67	0.86	1.02	0.23
Yangtze River ^2^	nd	0.2	1.7	0.1	nd	0.1
Amazon River ^2^	nd	nd	nd	0.8	nd	nd
Yellow River ^2^	nd	0.30–0.59	0.96–1.6	0.07–0.32	nd	0.01–4.1
Upper Mississippi ^2^	nd	1.66	1.85	0.12	1.11	0.01
World Average ^2^	0.70	0.80	1.48	0.04	0.42	0.08

^1^ nd: no data; ^2^ Dissolved trace element concentrations in the Yangtze, Amazon, Yellow, upper Mississippi River, and world average values were collected by Gaillardet et al. (2014) [1].

**Table 3:** Pearson correlation matrix of water parameters and dissolved heavy metals in the Lancangjiang River.

	T (°C)	pH	TDS	DO	Cr	Ni	Cu	Zn	Mo	Pb
T	1									
pH	−0.750 **	1								
TDS	−0.576 **	0.446 **	1							
DO	0.136	−0.012	−0.193	1						
Cr	0.184	−0.058	−0.249	−0.102	1					
Ni	−0.312 *	0.300 *	0.262	−0.098	−0.037	1				
Cu	0.205	−0.162	−0.035	−0.144	−0.063	0.296 *	1			
Zn	0.123	−0.350 *	−0.070	−0.054	0.040	−0.053	0.017	1		
Mo	−0.574 **	0.412 **	0.552 **	−0.269	−0.282	0.637 **	0.097	−0.076	1	
Pb	0.181	−0.059	−0.036	0.025	−0.146	−0.027	−0.190	0.046	−0.100	1

* strong correlation coefficients at 0.05 level (two-tailed), ** strong correlation coefficients at 0.01 level (two-tailed). Number of samples: n = 45.

**Table 4:** Varimax rotated component matrix for the dissolved heavy metals in the Lancangjiang River.

PC	1	2	3
Eigenvalues	1.881	1.217	1.003
Variance (%)	31.342	20.277	16.723
Cumulative (%)	31.342	51.619	68.342
Ni	0.880	0.089	0.039
Mo	0.855	−0.177	−0.117
Cu	0.478	0.458	0.062
Pb	−0.128	−0.753	0.173
Cr	−0.333	0.634	0.161
Zn	−0.013	−0.030	0.973

Extraction method: Principal component analysis. Rotation method: Varimax with Kaiser normalization. Rotation converges after five iterations. Number of samples: n = 45.

**Table 5:** Weights for the dissolved heavy metals in the Lancangjiang River.

	Eigenvalue (%)	Relative Eigenvalue ^1^	Variable	Loading Value	Relative Loading Value ^2^	W_i_^3^	Guidelines ^4^ (μg/L)
PC1	1.881	0.459	Ni	0.880	0.398	1.153	20
			Mo	0.855	0.386	1.187	70
			Cu	0.478	0.216	2.124	1000
			Total	2.213	1.000	0.459	
PC2	1.217	0.297	Cu	0.458	0.419	0.708	1000
			Cr	0.634	0.581	0.511	50
			Total	1.092	1.000	0.297	
PC3	1.003	0.245	Zn	0.973	1.000	0.245	1000
			Total	0.973	1.000	0.245	
Total	4.101						

^1^ Relative eigenvalues = Eigenvalues of each PC/Total Eigenvalues; ^2^ Relative loading values = Loading values of each variable/Total loading values of each PC; ^3^ Wi = Relative eigenvalues/Relative loading values; ^4^ guidelines were obtained from China GB 5749-2006 [42]. number of samples: n = 45.

## Supplementary Materials

The following are available online at https://www.mdpi.com/1660-4601/17/3/732/s1, Table S1. Hazard quotient and hazard index for dissolved heavy metals in the Lancangjiang River.
